# Overexpression of OsPT8 Increases Auxin Content and Enhances Tolerance to High-Temperature Stress in *Nicotiana tabacum*

**DOI:** 10.3390/genes10100809

**Published:** 2019-10-14

**Authors:** Zhaopeng Song, Ningbo Fan, Guizhen Jiao, Minghong Liu, Xiaoyan Wang, Hongfang Jia

**Affiliations:** 1 College of Tobacco Science, Henan Agricultural University, Zhengzhou 450002, China; ycszp@henau.edu.cn (Z.S.); 13643812806@163.com (N.F.); jiaoguizhen@henau.edu.cn (G.J.); 2 Zunyi Branch of Guizhou Tobacco Company, Zunyi 563000, China; lmh859@163.com (M.L.); xiaoyan8210@163.com (X.W.)

**Keywords:** *OsPT8*, high-temperature stress, root architecture, auxin, *Nicotiana tabacum*

## Abstract

Temperature is a primary factor affecting the rate of plant development; as the climate warms, extreme temperature events are likely to increasingly affect agriculture. Understanding how to improve crop tolerance to heat stress is a key concern. Wild plants have evolved numerous strategies to tolerate environmental conditions, notably the regulation of root architecture by phytohormones, but the molecular mechanisms of stress resistance are unclear. In this study, we showed that high temperatures could significantly reduce tobacco biomass and change its root architecture, probably through changes in auxin content and distribution. Overexpression of the *OsPT8* phosphate transporter enhanced tobacco tolerance to high-temperature stress by changing the root architecture and increased the antioxidant ability. Molecular assays suggested that overexpression of *OsPT8* in tobacco significantly increased the expression of auxin synthesis genes *NtYUCCA 6, 8* and auxin efflux carriers genes *NtPIN 1,2* under high-temperature stress. We also found that the expression levels of auxin response factors *NtARF1* and *NtARF2* were increased in *OsPT8* transgenic tobacco under high-temperature stress, suggesting that *OsPT8* regulates auxin signaling in response to high-temperature conditions. Our findings provided new insights into the molecular mechanisms of plant stress signaling and showed that *OsPT8* plays a key role in regulating plant tolerance to stress conditions.

## 1. Introduction

Temperature is one of the most important factors affecting plant growth and development [1,2,3,4], and with increasing global temperatures, the ability of plants to tolerate high-temperature stress is a key concern for agriculture [5]. High temperatures inhibit a series of physiological processes in plants, including photosynthesis and respiration, and have a negative impact on crop production and quality [6,7,8]. To survive under high-temperature stress conditions, plants have evolved various tolerance strategies, including increased scavenging capacity of reactive oxygen species (ROS) [8], rapid gene promotion, and modified root architecture [9,10]. Although the impact of high-temperature stress on plant growth has been documented, the mechanisms regulating this outcome are poorly understood.

Arguably the most important plant hormone, auxin (IAA, indole-3-acetic-acid), plays a role in cell division and elongation, gametogenesis, and embryo development [11,12]. Previous studies have shown that auxin plays an important role in regulating plant responses to environmental stress, for example, changing the root architecture to cope with high-temperature stress in *Arabidopsis* [13,14,15]. Until now, a lot of studies showed that some auxin-related genes (*PINs* and *AUXs*) involve in regulating root growth and development under high-temperature stress conditions [16,17,18]. Further, the introduction of exogenous auxin can reverse male sterility caused by high temperatures [19]. Recently, Zhang et al (2017) reported that auxin might prevent the degradation of spikelets in rice plants by mediating the salicylic acid pathway [20]. This evidence suggests that auxin can be used to mitigate the negative effects of high-temperature stress. Developing a comprehensive understanding of auxin synthesis and transport in plants under high-temperature stress is key to securing agricultural productivity in regions experiencing climate warming.

Phosphorus (P) is one of the major macronutrients involved in plant growth and development [21,22]. Inorganic phosphate (Pi) is taken up by plant roots via Pi transporters [23]; a large number of Pi transporter genes have been identified across different plant species and are generally classified into the *Pht1*, *Pht2*, *Pht3*, and *PT* gene families [24]. The *Pht1* family plays a crucial role in both Pi uptake and translocation under Pi deficiency [25,26]. Previous studies have shown that *OsPT8* is a high-affinity phosphate transporter and plays a key role in the Pi homeostasis in rice [22]. Moreover, *OsPT8* is not only involved in Pi signaling but also auxin signaling [27]. Notably, overexpression of *OsPT8* in rice and tobacco plants changes the root architecture using the auxin signaling pathway; it also regulates disease resistance and growth in rice, implying that *OsPT8* may play a key role in plant growth, development, and tolerance to stress [28,29,30]. Although the function of *OsPT8* in Pi uptake and transport has been documented, the molecular mechanisms which regulate environmental stress in plants are still unclear.

Tobacco is a model plant and an economically important crop [31,32,33]. In this study, we aimed to clarify how high-temperature stress affects the function of *OsPT8*, using tobacco plants as the study system. We used *DR5::GUS*, *OsPT8*-overexpression, and *DR5::GUS*/*OsPT8*-overexpression transgenic tobacco plants to study: (1) auxin content and distribution in tobacco plants under high-temperature conditions and (2) the function of *OsPT8* in enhancing high-temperature tolerance in tobacco through regulation of the auxin signaling pathway. This study explored the potential of *OsPT8* as a candidate gene for improving plant resistance to high-temperature stress.

## 2. Materials and Methods

### 2.1. Plant Materials, Growth Conditions, and Stress Treatments

The DR5::GUS, *OsPT8*-overexpression, and DR5::GUS/*OsPT*8-overexpression transgenic tobacco plants and wild type (*Nicotiana tabacum cv*, *Yunyan 87*) were used in this study. The generations of transgenic plants by *Agrobacterium-*mediated transformation, as described previously [27,28,29].

The 150–200 tobacco seeds of WT and *OsPT8*-Oe transgenic plants were sterilized in solution of 75% (v/v) ethanol for 30 s and 10% (v/v) sodium hypochlorite for 10 min, then followed by washing 6 times with sterile distilled water, and the seeds were then transferred to seedling tray (3 days) kept in culture room at 27 °C in dark environment for proper germination. Germinated seedlings were placed in light incubators (RXZ-600; Ningbo Jiangnan Instrument Factory, Ningbo City, China) with a 14-h-light/10-h-dark photoperiod and a day/night temperature of 27 °C/22 °C, and the relative humidity was controlled at approximately 60%, and a light intensity of 300 μmol·m^-2^·s^-1^ for 7 days. Sixteen tobacco seedlings were grown in each culture vessel (Diameter: 15 cm; Depth: 5 cm) with sands, and the Hoagland’s nutrient solution was changed every day in the growth chamber (described above). In the first 3 days, it was cultured using 1/4 strength Hoagland’s nutrient solution, and 1/2 strength nutrient solution was used in the second 3 days. Then, the seedlings were transferred to the full-strength culture solution for the next experiment.

For high-temperature stress treatments, some seedlings of wild type and transgenic tobacco were transferred to the light incubators with high-temperature (a day/night temperature of 37 °C/32 °C), which also has the same photoperiod, relative humidity, and light intensity with control treatment (a day/night temperature of 27 °C/22 °C). The plants were treated with high-temperature for three weeks, and then the tobacco seedlings were harvested. The collected plants were washed with deionized water for further analysis. (1) Observed and recorded the phenotype of tobacco plants. (2) Some seedlings were used to measure the IAA, chlorophyll, and MDA (malondialdehyde) content. (3) Some of the leaves and roots were used to NBT (nitroblue tetrazolium) and *DR5::GUS* staining. (4) The other tobacco seedlings were used to measure the Pi contents. (5) Some seedlings were used for detecting the gene expression and enzyme activity.

For exogenous IAA (indole-3-acetic acid dissolved in 1M NaOH) treatment, the 100 nM IAA was added to the nutrient solution under high-temperature treatments. The experimental design included four treatments: 27 °C, 27 °C + IAA, 37 °C, 37 °C + IAA. The tobacco seedlings were grown in a growth chamber for 7 days. Then, the plants were harvested for the next analysis. (1) To observe the phenotype of tobacco plants. (2) To record the histochemical localization of GUS.

### 2.2. GUS Staining and Nitroblue Tetrazolium (NBT) Staining

The tobacco seedlings were stained for GUS activity for 24 h at 37 °C, and then samples were immersed in 95% ethanol to eliminate chlorophyll pigmentation. For nitroblue tetrazolium (NBT) staining, the tobacco seedlings were stained with NBT solution for 5 h at 30 °C, and then 95% ethanol was used until decolorization was complete. The stereo microscope (Olympus SZX16, Olympus, Tokyo, Japan) equipped with a color CCD camera was used to photograph stained plant tissues.

### 2.3. Chlorophyll, MDA, Proline, and Pi Measurement

The relative amount of chlorophyll in plants was determined by a SPAD-502 chlorophyll meter (SPAD-502; Konica, Minolta Sensors, Japan) [34]. Five sites of one leaf were measured, and the results were averaged. The content of malondialdehyde (MDA) in plants was measured by thiobarbituric acid (TBA) method [35], and the content of free proline (Pro) in plants was determined by the sulfosalicylic acid method [36]. Inorganic phosphorus (Pi) content in plants was determined by the molybdenum blue method, as described previously [23].

### 2.4. IAA Measurement

The IAA contents of shoots and roots in tobacco seedlings were measured, as described by Sun et al. (2014) and Jia et al. (2018) [29,37]. A sample of 0.2 g was ground with an appropriate amount of the antioxidant butylated hydroxytoluene (BHT) and 80% pre-cooled methanol for 12–16 h. The extracted fluid was collected and concentrated by a rotary evaporator to 10 mL at 40 mL, and then the fluid was extracted with petroleum ether of the same volume. Underlayer, the liquid was adjusted to pH 8.5, and 0.2 g polyvinylpyrrolidone (PVP) was added, then vibrated for 30 min, and then filtered through a 0.45 μm filter over an OASIS HLB (St. Louis, MO, USA), and chromatographic conditions were described by Waters 600-2487; Hibar column RT 250 × 4.6 mm; Purospher STARRP-18 (5 μm); column temperature 45 °C; fluid phase: methanol:1% acetic acid (v/v, 40/60), isocratic elution; fluid rate: 0.6 mL min^-1^; UV detector, l = 269 nm; injection volume 20 μL. A 0.22 μm filter was used for filtration of both the buffer and the samples before HPLC analysis.

### 2.5. RNA Extraction, cDNA Synthesis, and RT-qPCR

Total RNAs were prepared from tobacco seedlings using Trizol reagent (Invitrogen, Carlsbad, CA, USA) according to the manufacturer’s instructions. The cDNA synthesis was done using the method described by Jia et al. (2018) [29]. Gene-specific primers were used for auxin-related genes (*YUCCAs*, *PINs*, and *ARFs* family genes) in tobacco by reverse transcription-polymerase chain reaction (RT-qPCR) analysis. The primers used for RT-qPCR are listed in Supplemental Table S1. The relative expression levels were normalized to that of *NtL25* (L18908.1) and presented as 2^−ΔΔCt^.

### 2.6. Statistical Analysis

All data were statistically analyzed via the SPSS 22.0 software by one-way ANOVA analysis, and subsequent multiple comparisons were performed based on the least significant difference (LSD) test; Statistical significance was set at * *p* < 0.05 and ** *p* < 0.01, and the charts were completed using the Origin 2018.

## 3. Results

### 3.1. IAA Alters Tobacco Root Architecture under High-Temperature Stress

**Figure 1:**
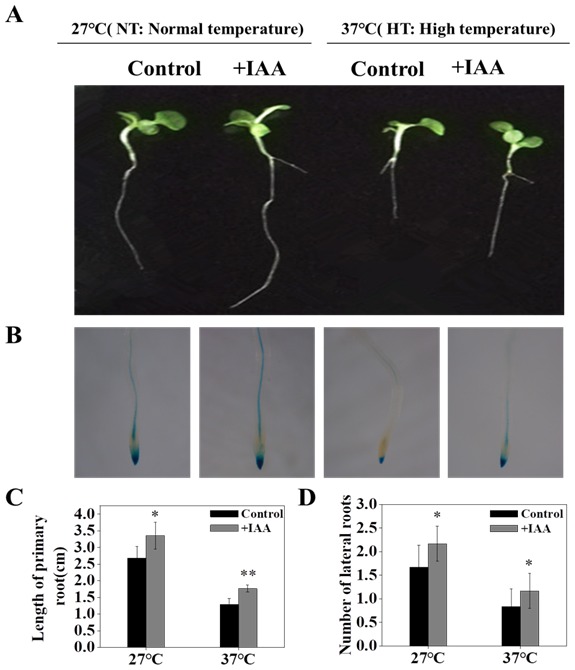
Characterization of root and GUS staining of *DR5::GUS* transgenic tobacco under high-temperature stress with adding IAA (indole-3-acetic-acid) (100 nM). (**A**) The phenotype of tobacco under high-temperature conditions with adding IAA. (**B**) Histochemical localization of *DR5::GUS* transgenic tobacco under high-temperature conditions with adding IAA. (**C**) Length of primary root under high-temperature conditions with adding IAA. (**D**) The number of lateral roots of tobacco under high-temperature conditions with adding IAA. 7-days-old seedlings were treated at different temperatures with adding IAA (100 nM) for 7 days. Control: no exogenous IAA added. Shown are mean ± SD from five independent biological replicates (*n* = 5). Level of significance: *p* < 0.05 *, *p* < 0.01 **.

To investigate the effect of IAA on high-temperature stress responses in tobacco, the length of the primary root and number of lateral roots were examined, and the IAA response was detected using GUS staining. Under high-temperature conditions, the length of the primary roots decreased significantly by 52.02%, and the number of lateral roots decreased by 50% compared to under normal temperature conditions (Figure 1C,D). This was consistent with the changes in IAA response, implying that IAA plays a key role in this process (Figure 1B). In addition, we found that the application of exogenous IAA caused a 37.35% increase in the length of primary roots and a 40% increase in the number of lateral roots (Figure 1A,C,D) under high-temperature conditions. Interestingly, we also found that the ratio of HT/NT (high-temperature/normal temperature) in length of primary and numbers of lateral roots had no significant difference with control under adding IAA conditions (Figure S1). Thus, it is likely that IAA promotes high-temperature tolerance partially through the auxin pathway.

### 3.2. The Expression of OsPT8 Is Upregulated under High-Temperature Conditions

**Figure 2:**
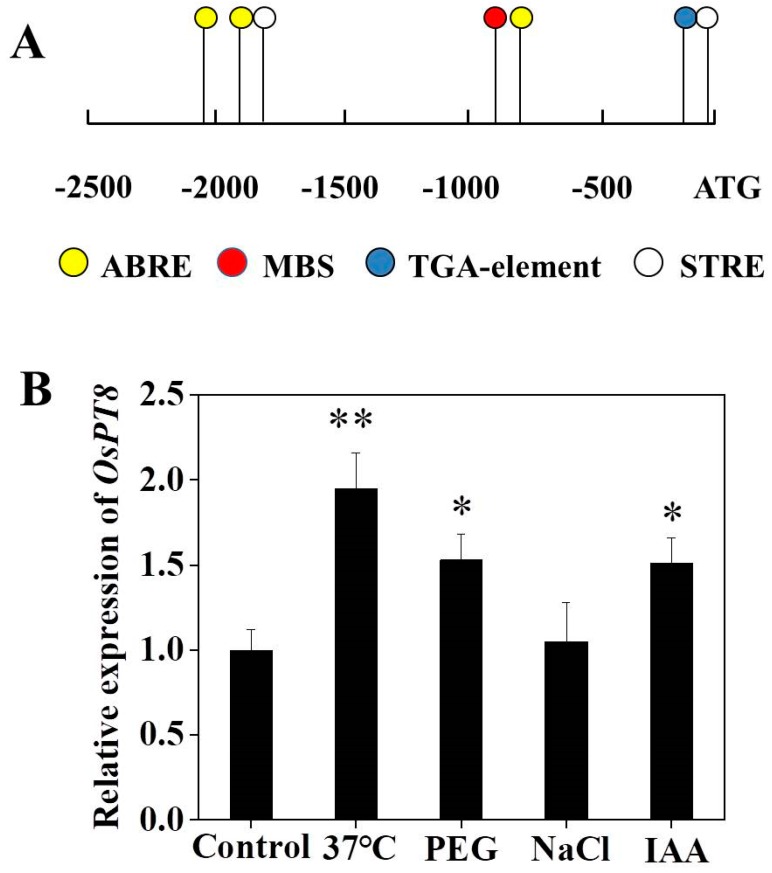
Analysis of an *OsPT8* promoter. **(A**) Stress-related motifs, such as ABRE (ACGTG), drought-associated element MBS (CAACTG), heat shock protein-related element STRE (AGGGG), and auxin response element TGA-element (AACGAC) in *OsPT8* promoter. (**B**) The expression levels of O*sPT8* under abiotic stress conditions. Fourteen-days-old seedlings were treated under different stress for 24 h. Control, under no stress. Shown are mean ± SD from five biological replicates (*n* = 5). Level of significance: *p* < 0.05 *, *p* < 0.01 **.

To determine whether *OsPT8* is regulated by abiotic stresses, the promoter sequence of *OsPT8* was analyzed using PlantCARE. Abiotic stresses-responsive elements, such as ABRE (ACGTG), drought-associated (CAACTG), and heat shock protein-related (AGGGG) elements, were detected in the *OsPT8* promoter, indicating that *OsPT8* might be involved in abiotic stress signaling in plants. We also identified an auxin response TGA-element (AACGAC) in the *OsPT8* promoter (Figure 2A). This suggests that *OsPT8* may be involved in auxin signaling pathways in plants. We further checked the expression levels of O*sPT8* under abiotic stress conditions, such as high-temperature, drought, and NaCl stress. The results indicated that *OsPT8* expression was significantly upregulated under high-temperature stress; however, almost no changes were observed under NaCl stress compared to control (under no stress conditions) (Figure 2B). We also found that the *OsPT8* expression was significantly upregulated by adding exogenous IAA (Figure 2B), which was consistent with the previous reports [27]. All these results implied that *OsPT8* might play a key role in facilitating the regulation of high-temperature stress responses by the auxin pathway.

### 3.3. Overexpression of OsPT8 Enhances High-Temperature Stress Tolerance in Tobacco

**Figure 3:**
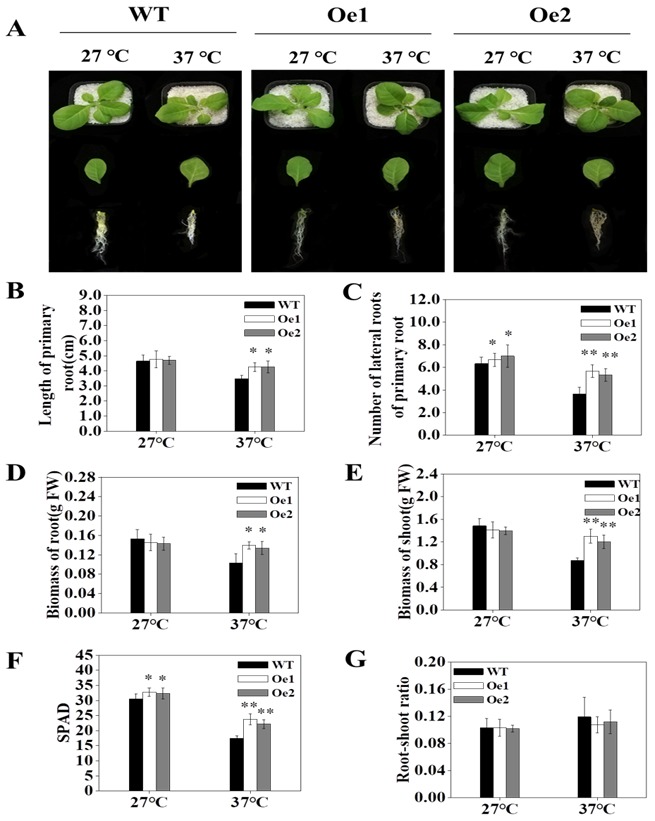
Phenotypic characteristics of tobacco under high-temperature stress for 3 weeks. (**A**) The characterization of tobacco under high-temperature treatments. (**B**) Length of primary root under high-temperature conditions. (**C**) The number of lateral roots of tobacco under high-temperature conditions. (**D**,**E**) The biomass of tobacco under high-temperature conditions. (**F**) The SPAD of the third young leaf of tobacco under high-temperature conditions. (**G**) The root/shoot ratio of tobacco under high-temperature conditions. Fourteen-days-old seedlings were treated at different temperatures (normal temperature: 27 °C; high-temperature: 37 °C) for 3 weeks. WT: wild-type, *Yunyan 87*; Oe1 and Oe2: transgenic tobacco. Shown are mean ± SD from five biological replicates (*n* = 5), and FW represents the fresh weight. Level of significance: *p* < 0.05 *, *p* < 0.01 **.

To determine the function of *OsPT8* on tobacco growth under high-temperature stress conditions, we measured the biomass of *OsPT8-Oe* transgenic tobacco and wild type (WT) under either NT (normal temperature: a day/night temperature of 27 °C/22 °C) or HT (high-temperature: a day/night temperature of 37 °C/32 °C) stress conditions. Under normal temperature, no significant difference in biomass was observed between WT and *OsPT8-Oe* tobacco plants. Notably, under high-temperature stress conditions, the *OsPT8-Oe* plants did not show significant symptoms of high-temperature stress (Figure 3A, Figure S2). For example, the leaves of these plants remained green, and their root and shoot biomass was significantly higher than those of WT plants (Figure 3D–F). Interestingly, the seeds germination rate of *OsPT8-Oe* tobacco was significantly higher than that of WT plants at 37 °C (Figure S3). Additionally, we detected the length of primary roots and the number of lateral roots in transgenic tobacco under high-temperature conditions. At 37 °C, the length of the primary roots and number of lateral roots of the transgenic tobacco plants increased by 22.11% and 50%, respectively, compared to WT plants (Figure 3B,C). Moreover, under high-temperature conditions, the root/shoot of transgenic plants did not significantly differ from WT plants (Figure 3G). These results indicated that overexpression of *OsPT8* in tobacco could promote root growth and development, and this might be the reason for the improved tolerance of *OsPT8*-Oe plants to high-temperature stress.

### 3.4. Overexpression of OsPT8 Enhances Antioxidant Capacity of Tobacco under High-Temperature Stress Conditions

**Figure 4:**
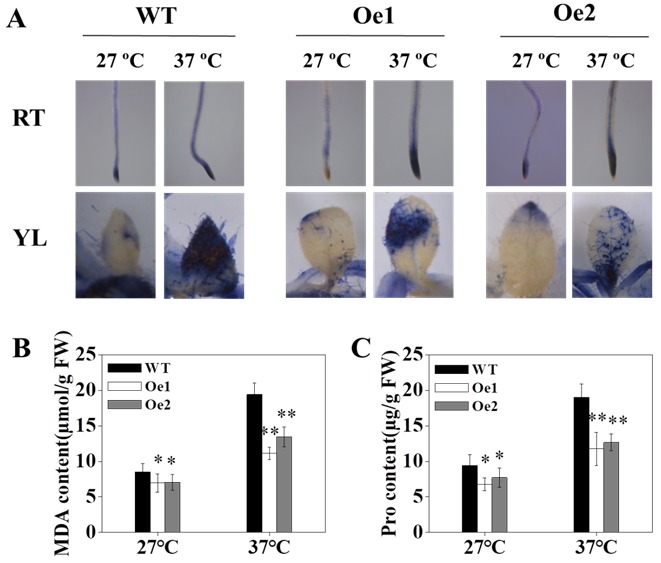
Effect of high-temperature stress on antioxidant capacity of tobacco. (**A**) NBT (nitroblue tetrazolium) staining of tobacco under high-temperature stress. (**B,C**) MDA (malondialdehyde) and Pro (proline) content under high-temperature conditions. Fourteen-days-old seedlings were treated at high temperatures for 3 weeks. Shown are mean ± SD from five biological replicates (*n* = 5). RT: root tip; YL: young leaf; FW, fresh weight. Level of significance: *p* < 0.05 *, *p* < 0.01 **.

To confirm the effect of *OsPT8* on tobacco antioxidant capacity under high-temperature stress conditions, we first used nitroblue tetrazolium (NBT) staining to determine the accumulation of H_2_O_2_. The staining results indicated that overexpression of *OsPT8* in tobacco markedly reduced the accumulation of H_2_O_2_ under high-temperature stress conditions (Figure 4A). We further evaluated the levels of MDA and Pro, which were found to be consistent with the accumulation of H_2_O_2_ (Figure 4B,C).

In addition, we also found that the contents of MDA and Pro in wild type tobacco increased by 1.28–fold and 1.02–fold under high-temperature conditions, respectively. However, compared with WT, the content of MDA and Pro in *OsPT8*-Oe transgenic tobacco only increased 0.76-fold and 0.69-fold, respectively (Figure 4B,C, Figure S4). Collectively, these results suggested that overexpression of *OsPT8* in tobacco promoted tobacco growth under high-temperature stress by improving the anti-oxidation activity of tobacco plants.

### 3.5. OsPT8 Is Involved in the Auxin Signaling Pathway of Tobacco under High-Temperature Stress Conditions

**Figure 5:**
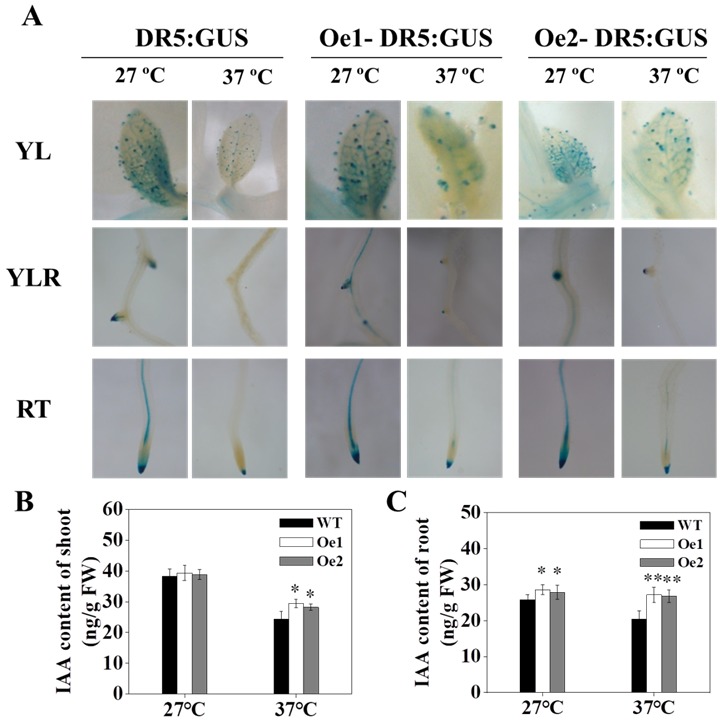
Histochemical localization of DR5::GUS and IAA contents of tobacco under high-temperature conditions. (**A**) GUS staining of tobacco under high-temperature conditions. (**B**,**C**) IAA content of the shoots and roots under high-temperature conditions. Fourteen-days-old tobacco seedlings (*DR5::GUS* transgenic tobacco) were treated at high temperatures for 3 weeks. Shown are mean ± SD from five biological replicates (*n* = 5). RT: root tip; YLR: young lateral root; YL: young leaf; FW: fresh weight. Level of significance: *p* < 0.05 *, *p* < 0.01 **.

The *DR5::GUS* reporter system can be used to study auxin distribution in plants [38]. To investigate whether *OsPT8* is involved in the auxin-induced growth of tobacco roots under high-temperature, we used *OsPT8-Oe* transgenic tobacco with a *DR5::GUS* reporter gene to analyze auxin distribution in tobacco plants. Our results demonstrated that the IAA content of tobacco root tips and leaves significantly decreased at high temperatures; however, the overexpression of the phosphorus transporter, *OsPT8*, could reverse this effect (Figure 5A–C). This suggested that *OsPT8* could increase the tolerance of tobacco plants to high-temperature stress by increasing IAA content.

**Figure 6:**
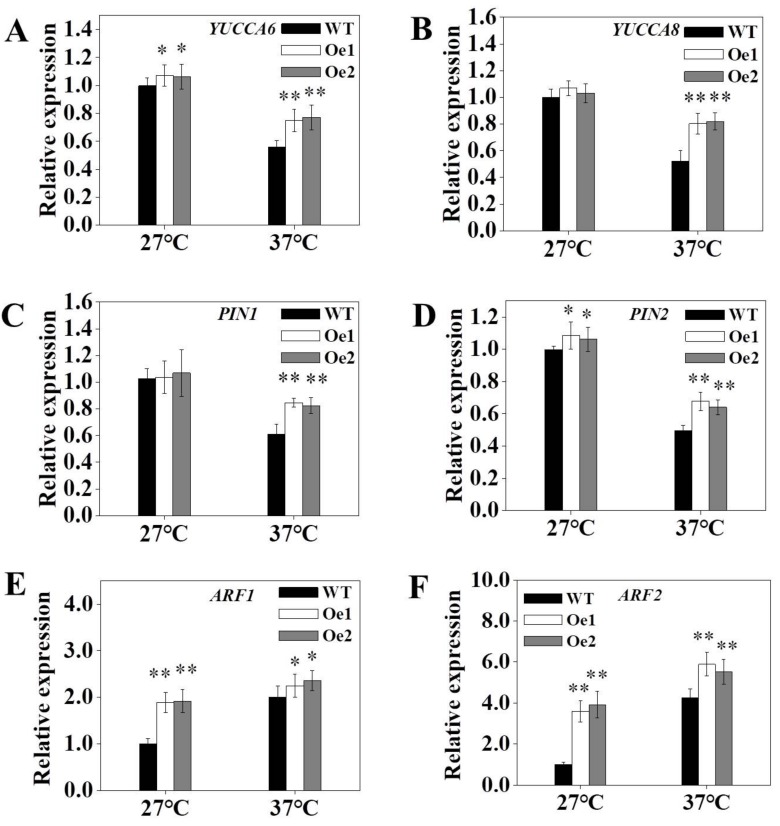
Expression of genes involved in auxin synthesis and transport under high-temperature conditions. (**A,B**) Expression of *YUCCAs* (*YUCCA6, 8*) family under high-temperature conditions. (**C,D**) Expression of *PINs* (*PIN1, 2*) family under high-temperature conditions. (**E,F**) Expression of *ARFs* (auxin response factors) (*ARF1, 2*) family under high-temperature conditions. *L25* was used as an internal control. Fourteen-days-old seedlings were treated at high temperatures for 3 weeks. Shown are mean ± SD from five biological replicates (*n* = 5). Level of significance: *p* < 0.05 *, *p* < 0.01 **.

Auxin levels are determined by a plant’s rate of auxin biosynthesis and transport capacity. To investigate the effect of *OsPT8* on the auxin signaling pathway, we analyzed the expression of key genes regulating IAA biosynthesis and transport under high-temperature stress. The results obtained indicated that the expression of *NtYUCCA6, 8* and *NtPIN1, 2* in transgenic tobacco was significantly higher than WT tobacco (Figure 6A–D). Moreover, analysis of the expression of auxin response factor (*ARF*) family genes, *ARF1* and *ARF2*, in transgenic tobacco, revealed that their expression was significantly upregulated compared to WT under high-temperature conditions (Figure 6E,F). Altogether, these results demonstrated that the upregulation of auxin-related genes promoted IAA synthesis and transport, which might modify root development under high-temperature stress conditions.

## 4. Discussion

Understanding how plants sense and respond to environmental temperatures is crucial to securing a future for agriculture under climate change [39,40]. Recent studies have shown that various genes and pathways are involved in the mechanisms by which plants sense and respond to ambient temperature [41,42,43]. In our work, we showed that the phosphate transporter *OsPT8* played an essential role in the regulation of auxin signaling and enhanced tobacco tolerance to high-temperature stress.

Auxin can act as a signaling molecule in plants to mediate physiological and biochemical processes, including root development and tissue differentiation [44,45,46]. Increasing temperatures inhibit the expression of IAA synthesis genes, downregulating IAA production [47,48]. In this study, we found that temperature stress significantly reduced the IAA content of tobacco roots and leaves, which resulted in changing plant root architecture (reduced length of the primary root and numbers of lateral roots), and affected plant growth and development (Figure 1 and Figure 5). Under high-temperature stress conditions, the expression levels of IAA-related genes, particularly *YUCCAs* and *PINs*, were lower than under normal temperature conditions (Figure 6A–D). Moreover, the expression of tobacco auxin response factors (*ARFs*), *ARF1* and *ARF2,* in roots increased (Figure 6E,F), indicating that high-temperature environments may affect auxin signaling, biosynthesis, and transport.

*OsPT8* plays a key role in Pi uptake and translation in rice. Previous studies showed the *OsPT8* expression in roots and shoots was upregulated distinctly under Pi deprivation [23,27]. In this work, we further analyzed the promoter sequence of *OsPT8* and detected the *OsPT8* expression level under high-temperature, NaCl, drought stress conditions in rice. The results showed that the expression of *OsPT8* also was upregulated under high-temperature stress conditions, suggesting that *OsPT8* might be involved in enhancing the tolerance to high-temperature stress (Figure 2). In addition, we further detected the expression of *NtPT1* and *NtPT2* in tobacco under high-temperature, NaCl, and drought stress conditions, which were consistent with the expression of *OsPT8* in rice, implying that overexpression of *OsPT8* in tobacco enhanced the tolerance to high-temperature stress by regulating other high-affinity Pi transporter in tobacco (Figure S5) [28]. Recently, Jia et al. (2017) reported that *OsPT8* also plays a key role in cross-talk between Pi and auxin signaling [27]. In this study, our results showed that overexpression of *OsPT8* in tobacco significantly increased the IAA content of roots and shoots under high-temperature stress compared with the WT, which was consistent with the expression levels of auxin-related genes—*ARF*s, *YUCCA*s, and *PIN*s—suggesting that *OsPT8* enhanced tobacco tolerance to high-temperature stress by increasing IAA content and modifying root growth (Figure 3, Figure 5 and Figure 6). This indicates that *OsPT8* could be a potential candidate gene for improving heat stress tolerance in tobacco, maize, and other crop plants, which might increase the yield of crops.

High-temperature stress destroys the membrane structure of cells, accumulates active oxygen, and accelerates cell senescence [49]. Previous studies have shown that the application of phosphorus can alleviate these negative effects in rice [50], implying a cross-communication between P-nitration signaling and ROS signaling. In this work, we confirmed that the accumulation of ROS significantly increased under high-temperature stress in tobacco. We also showed that overexpression of *OsPT8* in tobacco significantly reduced the accumulation of ROS compared with the WT under high-temperature conditions, which might be caused by increased antioxidant enzyme activity and elevated Pi content (Figure 4, Figure S6).

**Figure 7:**
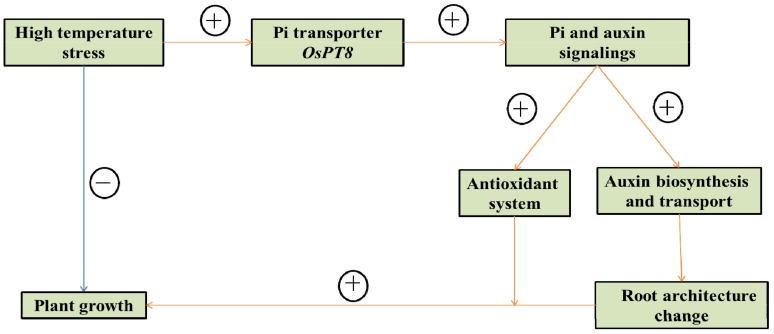
The model for the interaction mechanism between high-temperature stress, Pi, and auxin response in tobacco. The model is based on the results provided by this experiment. + indicates positive regulation, − indicates negative regulation.

Based on our findings, we proposed a possible model for the interaction mechanism between P signaling, auxin signaling, and high-temperature stress response in tobacco (Figure 7). Under high-temperature stress conditions: (1) wild type tobacco growth is inhibited, with decreased length of the primary root and fewer lateral roots; (2) *OsPT8* overexpression increases the expression of auxin-related genes, which increases IAA content, changes root architecture, and increases root biomass; (3) the *OsPT8* overexpression also enhances the antioxidant capacity of tobacco. Therefore, overexpression of *OsPT8* promotes plant growth under high-temperature stress conditions.

## 5. Conclusions

Taken together, our results indicate that *OsPT8*, a phosphate transporter, was involved in abiotic stress signaling by the auxin pathway in the plant. Overexpression of the *OsPT8* enhanced tobacco tolerance to high-temperature stress by affecting the auxin concentration and antioxidant capacity. The results showed that *OsPT8* might be a potential candidate gene for breeding plant species that can withstand high-temperature in plants, which would provide a theoretical basis to high yield of crops.

## Supplementary Materials

The following are available online at https://www.mdpi.com/2073-4425/10/10/809/s1, Table S1: The accession numbers and primers used in this work, Figure S1: Effects of exogenous IAA on the root architecture of tobacco under high temperature stress, Figure S2: Phenotypic characteristics of tobacco under high-temperature stress. Seven-days-old seedlings were treated at high temperatures for one week, and the effects of high-temperature on tobacco seedlings were illustrated by photographs, Figure S3: Seed germination rate under high-temperature conditions, Figure S4: Effect of high-temperature stress on antioxidant capacity of tobacco, Figure S5: The expression levels of *NtPT*s under abiotic stress conditions, Figure S6: The Pi content under high-temperature stress conditions.
