# Supplementary material for: Overexpression of OsPT8 Increases Auxin Content and Enhances Tolerance to High-Temperature Stress in Nicotiana tabacum
Source: Genes (Basel). 2019 Oct 14;10(10):809. doi: 10.3390/genes10100809 (PMC6826746; doi:10.3390/genes10100809)
Supplement: Supplementary file 1 [file genes-10-00809-s001.pdf]

SUPPORTING DATA:

Table S1. The accession numbers and primers used in this work.

Gene	No. ID	Primer Sequence (5' to 3')
OsPT8-Oe	AF536968	**F:**AATTGTTAACATGGCGCGGCAGGAGCAGC<br>**R:**AATTCTCGAGCTACGCCGTCTGCGGCCG
OsPT8	AF536968	**F:**GCAGATGGTGACCCGGAACAGC<br>**R:**CGAGGAACGTGCAGATGAACCC
ARF1	XM_016609214	**F:**CGGATCATCACATCATCAGC<br>**R:**TGGGAAATAGTAAACTCTCT
ARF2	XM_016621326	**F:**CGGCAAATCACTTCGGTGGC<br>**R:**ATAAACTCTTTCCCCTTCAC
NtPIN1	KC347302.1	**F:**GGAGCTGCAGCACAACAAAGT<br>**R:**ACCTTTCTTGTTATTAGTGC
NtPIN2	KP143726.1	**F:** TGCAATTATACCATTATATG<br>**R:** AAACCAAGCAATGGAACGGC
YUCCA6	XM_019373868	**F:** GGGTCCAGTAATTGTAGGAGC<br>**R:** TTTGAGTTGCCATAAAGAAGC
YUCCA8	XM_016592388	**F:**ATGTGTATGGGTAAATGGTCC<br>**R:**CAGATTTTTCCAAGATTACAC
NtL25	L18908	**F:**CCGTCCAAAAAATCTGACCC<br>**R:**TCTTCAAAGTCTTAGGTCGG

Figure S1.

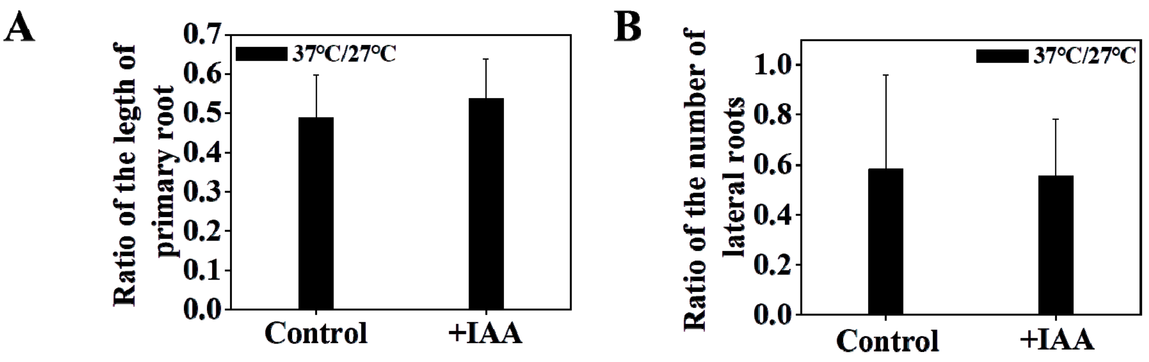

Figure S1. Effects of exogenous IAA on the root architecture of tobacco under high temperature stress. (A) Ratio of the length of primary root. (B) Ratio of the number of lateral roots. 7-days-old seedlings were treated at high temperatures for one week, the effects of high temperature on tobacco seedlings were illustrated by photographs. Shown are mean ±SD from five biological replicates (n = 5). Level of significance: P < 0.05 *, P < 0.01 **

Figure S2.

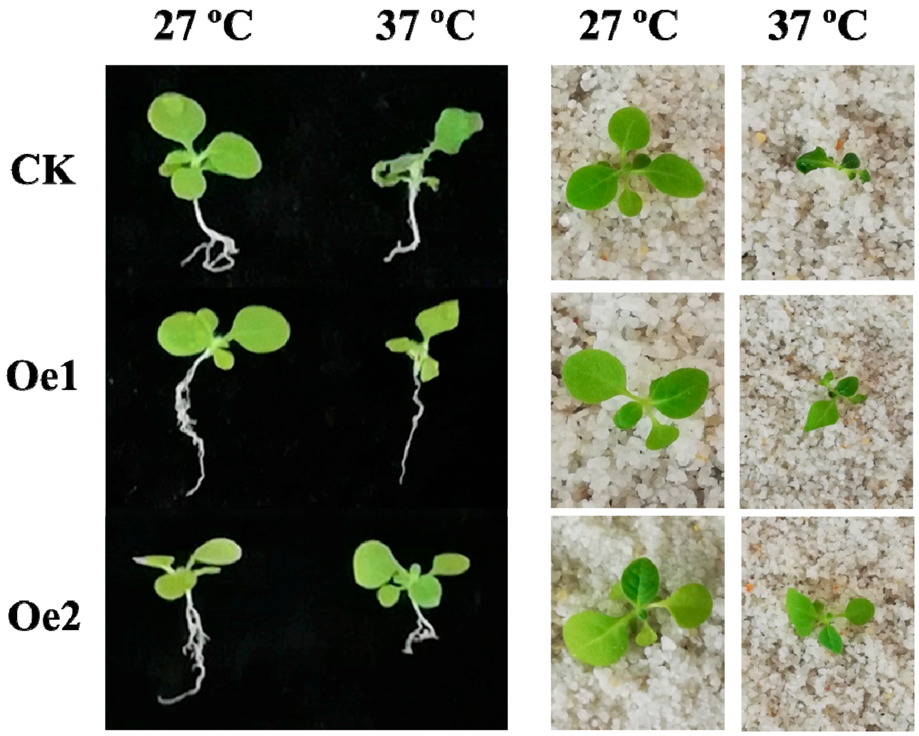

Figure S2. Phenotypic characteristics of tobacco under high temperature stress. 7-days-old seedlings were treated at high temperatures for one week, the effects of high temperature on tobacco seedlings were illustrated by photographs.

Figure S3.

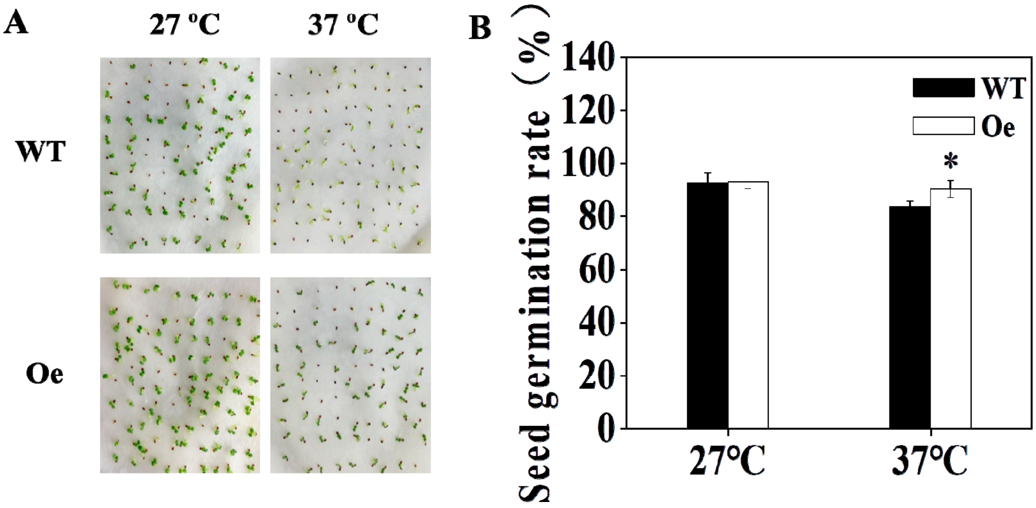

Figure S3. Seed germination rate under high temperature conditions. The seeds were treated for one week, each 200 tobacco seeds of WT and *OsPT8*-Oe transgenic plants were used and the germination rate was recorded. Shown are mean ±SD from five biological replicates (n = 5). Level of significance: P < 0.05 *, P < 0.01 **

Figure S4.

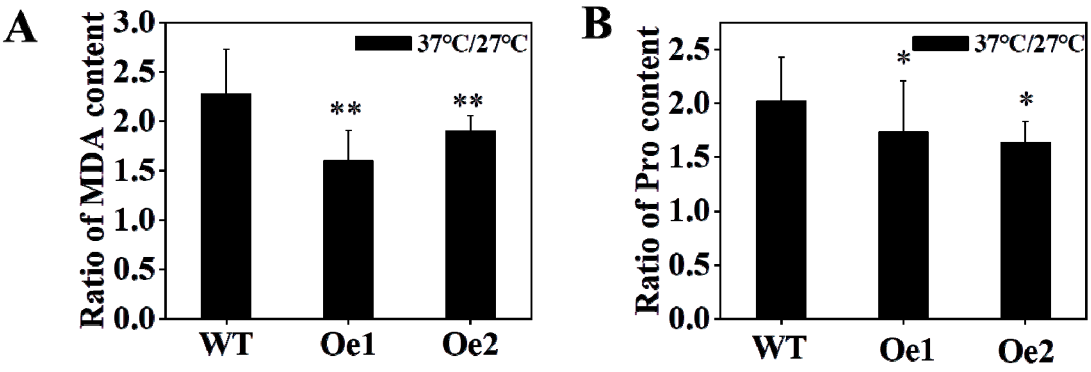

Figure S4. Effect of high temperature stress on antioxidant capacity of tobacco. (A) Ratio of MDA content under high temperature conditions. (B) Ratio of Pro content under high temperature conditions. The seedlings were treated at high temperatures for 3 weeks, shown are mean ±SD from five biological replicates (n = 5). Level of significance: P < 0.05 *, P < 0.01 **.

Figure S5.

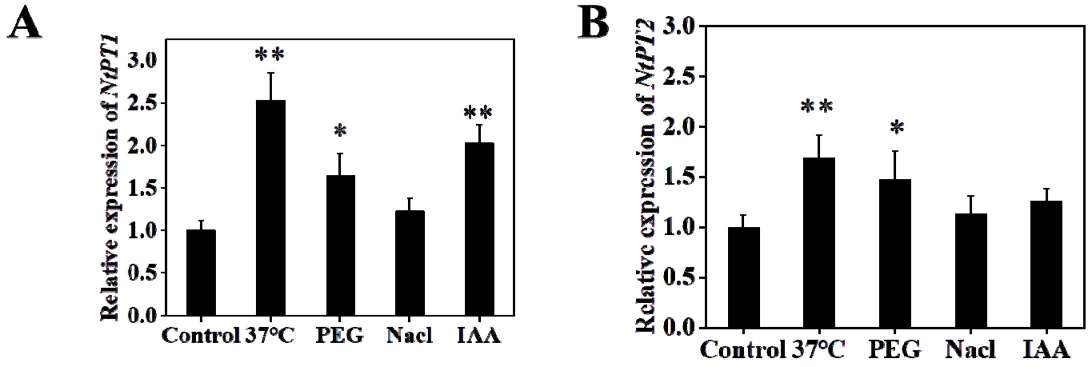

Figure S5. The expression levels of NtPTs under abiotic stress conditions. (A) Expression of *NtPT1*; (B) Expression of *NtPT2*. L25 was used as an internal control. Control, under no stress. 14-days-old seedlings were treated at high temperatures for 3 weeks. Shown are mean ±SD from five biological replicates (n = 5). Level of significance: P < 0.05 *, P < 0.01 **.

Figure S6.

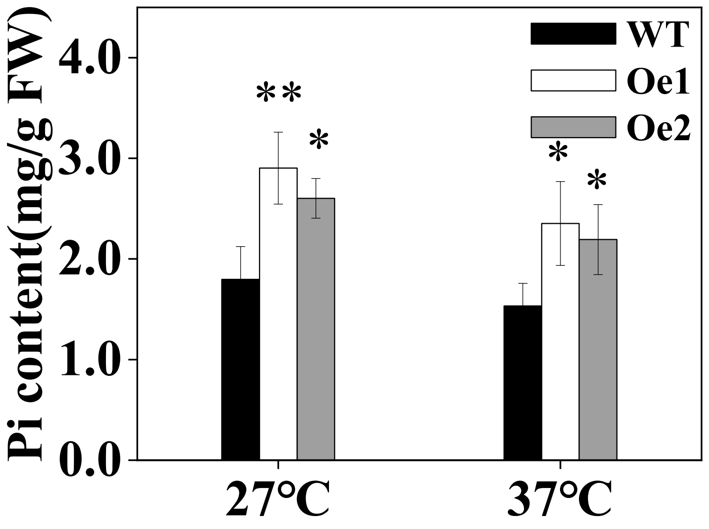

Figure S6. The Pi content under high temperature stress conditions. 14-days-old seedlings were treated at high temperatures for 3 weeks. Shown are mean ±SD from five biological replicates (n = 5). Level of significance: P < 0.05 *, P < 0.01 **
